# The Rapid Atrial Swirl Sign for Ultrasound-Guided Tip Positioning of Retrograde-Tunneled Hemodialysis Catheters: A Cross-Sectional Study from a Single Center

**DOI:** 10.3390/jcm10173999

**Published:** 2021-09-03

**Authors:** Peter Korsten, Tim Kuczera, Manuel Wallbach, Björn Tampe

**Affiliations:** Department of Nephrology and Rheumatology, University Medical Center Göttingen, 37075 Göttingen, Germany; peter.korsten@med.uni-goettingen.de (P.K.); tim.kuczera@med.uni-goettingen.de (T.K.); manuel.wallbach@med.uni-goettingen.de (M.W.)

**Keywords:** rapid atrial swirl sign, retrograde-tunneled hemodialysis catheter, ultrasound-guided tip positioning, vascular access, end-stage renal disease, renal replacement therapy

## Abstract

Background: Chronic kidney disease (CKD) is a common medical problem in patients worldwide, with an increasing prevalence of patients with end-stage kidney disease (ESKD) requiring renal replacement therapy (RRT). In patients requiring RRT for more than two weeks or those who develop ESKD, tunneled hemodialysis catheter (HDC) insertion is preferred, based on a lower risk for infectious complications. While the efficacy of ultrasound (US)-guided tip positioning in antegrade-tunneled HDCs has previously been shown, its application for the insertion of retrograde-tunneled HDCs has not been described yet. This is especially important, since the retrograde-tunneled technique has several advantages over the antegrade-tunneled HDC insertion technique. Therefore, we here report our first experience of applying the rapid atrial swirl sign (RASS) for US-guided tip positioning of retrograde-tunneled HDCs. Methods: We performed a cross-sectional study to assess the feasibility of applying the RASS for US-guided tip positioning of retrograde-tunneled HDCs. We performed a total number of 24 retrograde-tunneled HDC insertions in 23 patients (requiring placement of a HDC for the temporary or permanent treatment of ESKD) admitted to our Department of Nephrology and Rheumatology at the University Medical Center Göttingen, Germany. Results: The overall success rate of applying the RASS for US-guided tip positioning of retrograde-tunneled HDCs was 24/24 (100%), with proper tip position in the right atrium in 18/23 (78.3%), or cavoatrial junction in 5/23 (21.7%) when RASS was positive and improper position when RASS was negative in 1/1 (100%), confirmed by portable anterior-posterior chest radiography, with only minor post-procedural bleeding in 2/24 (8.3%). In addition, this insertion technique allows optimal HDC flow, without any observed malfunction. Conclusion: This is the first study to investigate the efficacy of the RASS for US-guided tip positioning of retrograde-tunneled HDCs in patients with ESKD. Application of the RASS for US-guided tip positioning is an accurate and safe procedure for the proper placement of retrograde-tunneled HDCs.

## 1. Introduction

Chronic kidney disease (CKD) is a common medical problem in patients worldwide, with an estimated global prevalence of 9.1% [1]. The prevalence of patients with end-stage kidney disease (ESKD) requiring renal replacement therapy (RRT) is estimated to increase and affect about 5.4 million people worldwide by the year 2030 [2]. Different modalities for RRT are available: peritoneal dialysis (PD), hemodialysis (HD), and kidney transplantation. The latter, however, is not immediately available for most patients. In addition, in an acute critical care setting, acute kidney injury (AKI) occurs in more than half of the patients admitted to an intensive care unit (ICU) and has an associated mortality of more than 50% [3]. Therefore, immediate short-term and intermediate-term vascular access needs to be established in this setting. Short-term, non-tunneled HD catheters (HDCs) bear the risk of infection, and their use for more than two weeks is discouraged by current guidelines [4]. As a consequence, in patients requiring RRT for more than two weeks or those who develop ESKD, tunneled HDC insertion is preferred, based on a lower risk for infectious complications as compared to non-tunneled HDCs [4]. The procedure is often performed by vascular surgeons or interventional nephrologists. Traditionally, it requires the use of fluoroscopic guidance to ensure correct placement and positioning of the tunneled HDC in the right atrium [4]. At our center, we have a dedicated ICU at our disposal, with a focus on critically ill patients with kidney diseases, rheumatic diseases, or related complications. Previously, we reported the feasibility and testing characteristics of ultrasound-guided assessment of correct central venous catheter (CVC) placement, using ultrasound (US)-guided tip positioning by an opacification of the right atrium after flush injection of saline immediately after catheter positioning, also known as the rapid atrial swirl sign (RASS) [5]. While the efficacy of US-guided tip positioning in antegrade-tunneled HDCs has previously been shown, application of the RASS for insertion of retrograde-tunneled HDCs has not been described, as of yet [6,7]. This is especially important because the retrograde-tunneled technique has several advantages over the antegrade-tunneled HDC insertion technique [8]. Here, we report our first experience of applying the RASS for US-guided tip positioning of retrograde-tunneled HDCs.

## 2. Materials and Methods

### 2.1. Study Population and Setting

We performed a retrospective cross-sectional study to assess the feasibility of applying the RASS for US-guided tip positioning of retrograde-tunneled HDCs. We included a convenience sample of patients who required the placement of a tunneled HDC for the temporary or permanent treatment of ESKD, admitted to our Department of Nephrology and Rheumatology at the University Medical Center Göttingen, Germany. We performed a total number of 24 retrograde-tunneled HDC insertions in 23 patients, from January 2020 to June 2021.

### 2.2. Catheter Placement Procedure and Material

For the placement of the retrograde-tunneled HDC, Palindrome™ Precision RT-reverse tunneled catheters (Medtronic, Minneapolis, MN, USA) were used. We used 15 french (F)-sized HDCs that were 23, 28, or 33 cm in length from tip to cuff, depending on the patient’s height and on the site of insertion (right or left). After obtaining informed consent by the patient, the procedure was performed by two interventionists, with continuous hemodynamic monitoring in a dedicated area of our ICU, to ensure maximum sterility and patient safety. The right internal jugular vein (IJV) was the preferred access site. After sterile preparation and draping, local anesthesia (with 2% mepivacaine hydrochloride) was applied, and the IJV puncture was performed, under US guidance (GE Venue US machine, General Electric Company, Boston, MA, USA), using a sterile probe cover, with an out-of-plane approach. After puncture, the guidewire was advanced into the vessel. Placement of the catheter was performed, according to the manufacturer’s instructions (an introductory video is freely available at https://www.youtube.com/watch?v=XIrc0hWIRJs, access date 30 June 2021). A subcutaneous tunnel for the catheter exit was prepared under local anesthesia.

### 2.3. Ultrasound Visualization and RASS

After placement of the retrograde-tunneled HDC, focused echocardiography by using subcostal (SC) and apical four-chamber (4C) or five-chamber (5C) views were used to visualize the right atrium. Echocardiography was performed using a sector probe of a GE Venue US machine (General Electric Company, Boston, MA, USA). Immediately after HDC placement, a flush (consisting of 10 mL of normal saline) was injected into one of the catheter hubs by one of the interventionists, while echocardiography was performed by a third operator. The exam was recorded in a short video sequence (on the hard disk of the US machine) for later review and documentation. The immediate appearance of the RASS, within one second, was judged as correct placement, as previously reported [5].

### 2.4. Post Procedural Assessment

After the placement and final positioning of the retrograde-tunneled HDC, conventional anterior chest radiography was performed to document the correct placement of the catheter tip and to exclude procedure-related complications. The patients were monitored for two hours following the procedure and transferred back to the ward for observation of potential complications for at least 24 h. Post-insertion HDC performance, including dialysis parameters, were assessed at first treatment performed the same or next day. 

### 2.5. Patient Consent and Ethics Approval

The study included patients aged >18 years of age who had an indication for HDC insertion. All patients provided written informed consent for all procedures presented in this paper, which are considered standard at our center. The study was conducted according to the guidelines of the Declaration of Helsinki and approved by the Ethics Committee of University Medical Center Göttingen (protocol number 3/6/21, approval date 25 June 2021).

### 2.6. Statistical Analysis

Descriptive statistics, with frequencies and percentages, were used for the characterization of the study cohort. No prespecified hypotheses were formulated, due to the exploratory nature of this study. Data analyses were performed with GraphPad Prism (version 9.1.1 for MacOS, GraphPad Software, San Diego, CA, USA).

## 3. Results

### 3.1. Study Population

Clinical parameters, including demographic data and etiology of ESKD in the total cohort of 23 patients, are included in Table 1.

**Figure 1:**
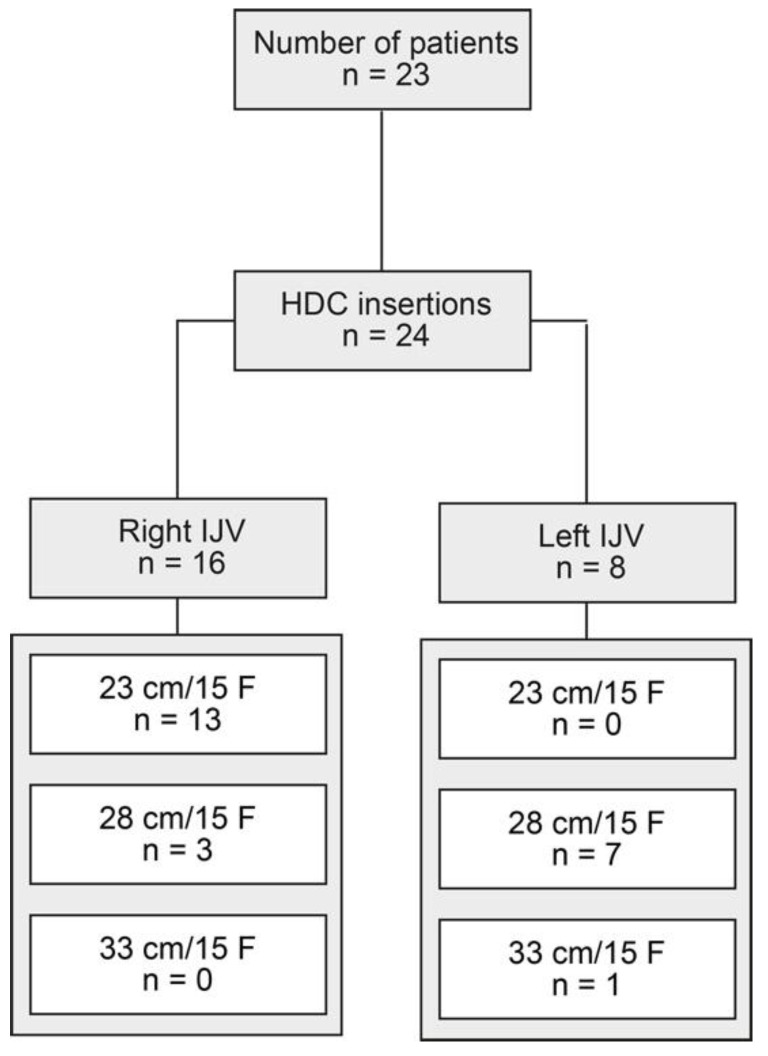
Total patient cohort. STROBE flow chart of patient disposition, with indication of retrograde-tunneled HDC insertion. Abbreviations: cm, centimeter; F, french; HDC, hemodialysis catheter; IJV, internal jugular vein; STROBE, Strengthening the Reporting of Observational Studies in Epidemiology.

We included a total number of 24 retrograde-tunneled, 15 F-sized HDC insertions in 23 patients (one HDC had to be replaced 45 days after the first insertion, due to HDC dislocation, Figure 1). A total of 16/24 (66.7%) retrograde-tunneled HDC insertions were performed through the right internal jugular vein (IJV), and 13/24 (54.2%) HDCs had a length of 23 cm (Figure 1). Laboratory data, at the time of 24 HDC insertions, are shown in Table 2. 

### 3.2. Application of RAAS for Retrograde-Tunneled HDC Tip Positioning

**Figure 2:**
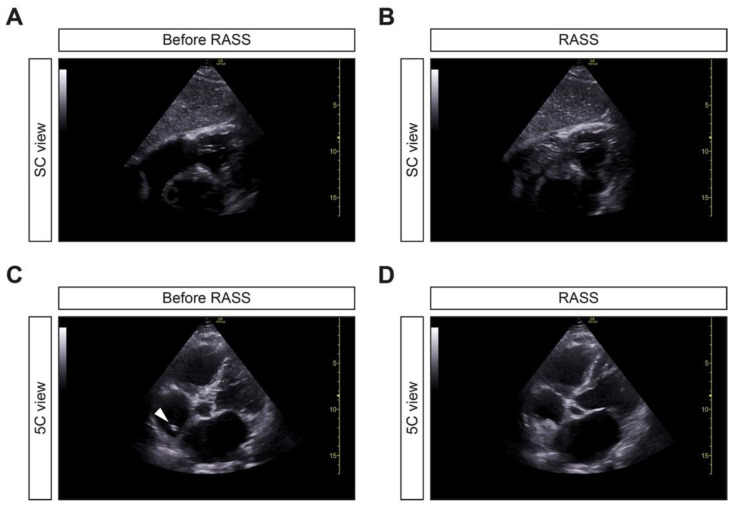
Application of the RASS for US-guided tip positioning of retrograde-tunneled HDCs. (**A**,**B**) After placement of the retrograde-tunneled HDC, focused echocardiography using a SC view to visualize the right atrium and RASS was performed. If the RASS appeared immediately, within one second, HDC tip positioning was considered to be adequate. (**C**,**D**) The HDC positioning was considered to be in the adequate position if the tip was visualized within the right atrium (arrowhead), with reconfirmed immediate RASS visualization in the apical 5C view, within one second. Abbreviations: HDC, hemodialysis catheter; RASS, rapid atrial swirl sign; SC, subcostal; US, ultrasound; 5C, five-chamber.

After the placement of the retrograde-tunneled HDC, focused echocardiography to visualize the right atrium and RASS was performed (Figure 2A,B). If the RASS appeared immediately, HDC tip positioning was considered to be adequate. If RASS visualization was delayed by more than one second, the HDC was considered to be inadequately positioned and repositioned. Thereafter, the HDC positioning was deemed to be in an adequate position if the tip was visualized within the right atrium with reconfirmed immediate RASS visualization in the apical 4C or 5C views (Figure 2C,D) [9]. Cine loop recordings (during the procedure) showing flow from the right atrium to the right ventricle, immediately after injection of 10 mL of normal saline, are shown (in SC and 5C views) in the Supplementary Video Files S1 and S2. RASS was positive in 23/24 (95.8%) and negative in 1/24 (4.2%) inserted retrograde-tunneled HDCs.

**Figure 3:**
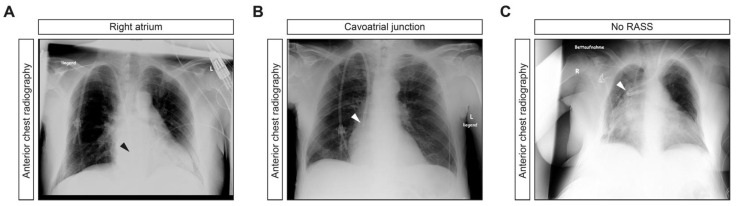
Portable anterior-posterior chest radiography. (**A**,**B**) Representative radiographic images of proper HDC placement, with tip position in the right atrium or cavoatrial junction (arrowheads). (**C**) Radiographic image of improper HDC placement, with kinking at the subclavian-caval junction, due to central vein stenosis (arrowhead). Abbreviations: HDC, hemodialysis catheter; RASS, rapid atrial swirl sign.

The overall success rate of applying the RASS for US-guided tip positioning of retrograde-tunneled HDCs was 24/24 (100%), with proper tip position in the right atrium in 18/23 (78.3%) or cavoatrial junction in 5/23 (21.7%) when RASS was positive and in improper position when RASS was negative, with kinking at the subclavian-caval junction, due to central vein stenosis in 1/1 (100%) HDC placements, confirmed by portable anterior-posterior chest radiography (Table 3 and Figure 3A–C).

### 3.3. Post-Procedural Complications after Retrograde-Tunneled HDC Placement

After HDC insertion, there were no post-procedural complications monitored for at least 24 h, despite minor post-procedural bleeding in 2/24 (8.3%) HDC insertions, requiring sandbag placement over the catheter insertion site (Table 4).

### 3.4. Post-Insertion HDC Performance

After HDC insertion, we monitored the dialysis parameters at first treatment, performed the same or next day, without any observed malfunction, a median blood flow of 200 mL/min, median dialysate flow of 500 mL/min, a median transmembrane pressure (TMP) of 40 mmHg, and median venous pressure of 100 mmHg (Table 5). In summary, we present the feasibility and safety of applying the RASS for US-guided tip positioning of retrograde-tunneled HDCs with optimal HDC flow, without any observed malfunction.

## 4. Discussion

To our knowledge, this is the first study performed in patients with ESKD to investigate the efficacy of the RASS for the US-guided tip positioning of retrograde-tunneled HDCs. In this cross-sectional study of our single center performed in patients requiring HDC insertion due to ESKD, we found that the application of the RASS for US-guided tip positioning was accurate in identifying the proper placement of retrograde-tunneled HDCs. The retrograde-tunneled technique has several advantages over the antegrade-tunneled HDC insertion technique [8]. First, the HDC tip position is established first and, thus, is consistent. Second, the cuff never passes through the exit site; therefore, only a small incision is required, which prevents bleeding and the accidental removal of the cuff before it is incorporated. Third, the cuff is placed at a distance of 2 cm from the marked exit site, which helps to avoid a cut-down when the catheter is removed. Finally, the hub is detachable and the replacement of a damaged hub or clamps is easy to perform without disturbing a functioning catheter. 

This is the first study of applying the RASS for the insertion of retrograde-tunneled HDCs in patients with ESKD. Traditionally, fluoroscopic guidance is used to ensure correct placement and positioning of the tunneled HDC in the right atrium [4]. The advantages of non-fluoroscopic methods for assessing the tip positiion of HDCs are obvious: avoiding radiation exposure, saving time, and faster possible use of the HDC. Multiple studies described the safety of US-guided tip positioning of CVCs without fluoroscopy [7,10]. The advantage of US-guided tip positioning is that it provides a dynamic procedure for catheter guidance, as well as the direct visual and functional assessment of the tip location. In addition, real-time fluoroscopy services are not always immediately available and impose additional safety risks to patients and operators, due to radiation exposure [11]. The US-guided insertion of CVCs was further improved by the use of different methods of flush injection and visualization of the right atrium, shown to be equally safe but faster and inexpensive [5,6,7]. The agitated, bubble-enhanced visualization is commonly prepared as a mixture of 9 mL of normal saline solution and 1 mL of air and has been shown to be a safe procedure, although rare events of ischemic cerebrovascular complications in patients with cardiac or intrapulmonary shunts have been reported and attributed to air bubbles [12,13]. In contrast, we have previously shown that the RASS provides an equally effective method for CVC insertion via the injection of 10 mL of normal saline, without the use of air; therefore, it is potentially safer, with regard to rare side effects [5]. Here, the overall success rate of applying the RASS for US-guided tip positioning of retrograde-tunneled HDCs was 100%, and there were no major adverse events with only minor post-procedural bleeding in 2/24 (8.3%) of HDC insertions. In addition, we show that this insertion technique allows for optimal post-procedural HDC flow, without any observed malfunction. 

Despite these observations, our study has several limitations. First, this is a cross-sectional study from a single center with a limited number of retrograde-tunneled HDC insertions. Second, all HDCs were inserted through the internal jugular veins and application to different access sites (e.g., external jugular veins, subclavian veins, and femoral veins) remains elusive. Third, our study did not intend to directly compare the application of the RAAS with the alternative use of fluoroscopy. Nevertheless, this is the first study to investigate the efficacy of the RASS for US-guided tip positioning of retrograde-tunneled HDCs as a feasible, safe, and easy procedure in interventional nephrology. Therefore, our observations require further corroboration in additional studies from other centers, to define the specific benefits and risks of exclusively US-guided procedures and the application of the RASS for retrograde-tunneled HDC tip positioning. Based on our observations, studies directly comparing RASS with fluoroscopy and applying the RASS for US-guided tip positioning of retrograde-tunneled HDCs without fluoroscopy would be of great interest. 

## 5. Conclusions

This is the first study to investigate the efficacy of the RASS for US-guided tip positioning of retrograde-tunneled HDCs in patients with ESKD. We describe that the application of the RASS for US-guided tip positioning is an accurate and safe procedure for the proper placement of retrograde-tunneled HDCs.

## Figures and Tables

**Table 1:** Clinical parameters in the total cohort of 23 patients.

**Demographic Data**	**Value**
Median age (IQR)—years	71 (56–76)
Female sex—no. (%)	7 (30.4)
Median height (IQR)—cm	172 (166–180)
Median BW (IQR)—kg	85 (75.5–104)
Median BMI (IQR)—kg/m^2^	30.5 (23.9–33.6)
History of previous catheterization—no. (%)	2 (8.7)
**Etiology of ESKD**	**Value**
Diabetic nephropathy—no. (%)	7 (30.4)
Cardiorenal syndrome—no. (%)	3 (13)
ANCA GN—no. (%)	2 (8.7)
Septic shock—no. (%)	2 (8.7)
Multiple myeloma/cast nephropathy—no. (%)	2 (8.7)
IgAN—no. (%)	1 (4.3)
Hypertensive nephropathy—no. (%)	1 (4.3)
Cardiogenic shock—no. (%)	1 (4.3)
Hemorrhagic shock—no. (%)	1 (4.3)
Chemotherapy nephrotoxicity—no. (%)	1 (4.3)
Fibrillary GN	1 (4.3)
ADPKD	1 (4.3)

Abbreviations: ADPKD, autosomal dominant polycystic kidney disease; ANCA, anti-neutrophil cytoplasmic antibody; BMI, body mass index; BW, body weight; cm, centimeter; ESKD, end-stage kidney disease; GN, glomerulonephritis; IgAN, IgA nephropathy; kg, kilograms; m, meter; IQR, interquartile range; no., number.

**Table 2:** Laboratory parameters at the time of 24 HDC insertions.

**Laboratory Data**	**Value**
Median platelet count (IQR)—×1000/μL	211 (108.3–317)
Median INR (IQR)—ratio	1.15 (1–1.2)
Median aPTT (IQR)—seconds	30 (28–34.3)
Hemoglobin—g/dL	8.4 (7.7–9.5)
**Vascular Access**	**Value**
Right IJV—no. (%)	16 (66.7)
Left IJV—no. (%)	8 (33.3)
**Catheter Length/Diameter**	**Value**
23 cm/15 F—no. (%)	13 (54.2)
28 cm/15 F—no. (%)	10 (41.7)
33 cm/15 F—no. (%)	1 (4.2)

Abbreviations: aPTT, activated partial thromboplastin time; cm, centimeter; F, french; GFR, glomerular filtration rate (CKD-EPI); HDC, hemodialysis catheter; IJV, internal jugular vein; INR, international normalized ratio; IQR, interquartile range; no., number.

**Table 3:** Position of retrograde-tunneled HDCs, assessed by portable anterior-posterior chest radiography, with regard to RASS (within one second) or no RASS.

Chest Radiography Position	RASS	No RASS
Right atrium—no. (%)	18 (75)	0 (0)
Cavoatrial junction—no. (%)	5 (20.8)	0 (0)
Subclavian-caval junction—no. (%)	0 (0)	1 (4.2)

Non-parametric between-group-comparisons were performed with Pearson’s Chi-square test. Abbreviations: no., number; HDC, hemodialysis catheter.

**Table 4:** Post-procedural complications after HDC placement, monitored at least 24 h.

Complications	Value
Arterial puncture—no. (%)	0 (0)
Pneumothorax—no. (%)	0 (0)
Hemothorax—no. (%)	0 (0)
Minor bleeding—no. (%)	2 (8.3)

Abbreviations: no., number; HDC, hemodialysis catheter.

**Table 5:** Retrograde-tunneled HDC performance data.

Dialysis Parameters	Value
Malfunction—no. (%)	0 (0)
Median blood flow (IQR)—mL/min	200 (200–250)
Median TMP (IQR)—mmHg	40 (20–60)
Median venous pressure (IQR)—mmHg	100 (80–120)

Abbreviations: IQR, interquartile range; HDC, hemodialysis catheter; TMP, transmembrane pressure. By facility praxis, dialysate flow rates were 500 mL/min.

## Data Availability

Deidentified data are available on reasonable request from the corresponding author.

## Supplementary Materials

The following are available online at https://www.mdpi.com/article/10.3390/jcm10173999/s1. Video files S1 and S2: demonstration of cine loop recordings during the procedure showing flow from the right atrium to the right ventricle immediately after injection of 10 mL of normal saline in SC and 5C views.

## References

[1] GBD Chronic Kidney Disease Collaboration (2020). Global, regional, and national burden of chronic kidney disease, 1990–2017: A systematic analysis for the Global Burden of Disease Study 2017. Lancet.

[2] Thurlow J.S., Joshi M., Yan G., Norris K.C., Agodoa L.Y., Yuan C.M., Nee R. (2021). Global Epidemiology of End-Stage Kidney Disease and Disparities in Kidney Replacement Therapy. Am. J. Nephrol..

[3] Griffin B.R., Liu K.D., Teixeira J.P. (2020). Critical Care Nephrology: Core Curriculum 2020. Am. J. Kidney Dis..

[4] Lok C.E., Huber T.S., Lee T., Shenoy S., Yevzlin A.S., Abreo K., Allon M., Asif A., Astor B.C., Glickman M.H. (2020). KDOQI Clinical Practice Guideline for Vascular Access: 2019 Update. Am. J. Kidney Dis..

[5] Korsten P., Mavropoulou E., Wienbeck S., Ellenberger D., Patschan D., Zeisberg M., Vasko R., Tampe B., Müller G.A. (2018). The “rapid atrial swirl sign” for assessing central venous catheters: Performance by medical residents after limited training. PLoS ONE.

[6] Passos R.D.H., Ribeiro M., da Conceicao L., Ramos J.G.R., Ribeiro J.C., Batista P.B.P., Dutra M.M.D., Rouby J.J. (2019). Agitated saline bubble-enhanced ultrasound for the positioning of cuffed, tunneled dialysis catheters in patients with end-stage renal disease. J. Vasc. Access.

[7] Elias R.M., Makida S.C.D.S., Abensur H., Castro M.C.M., Moyses R., Pereira B.J., De Oliveira R.B., Luders C., Egidio R.J. (2010). Insertion of Tunneled Hemodialysis Catheters without Fluoroscopy. J. Vasc. Access.

[8] Bream P.R. (2016). Update on Insertion and Complications of Central Venous Catheters for Hemodialysis. Semin. Interv. Radiol..

[9] Peritoneal Dialysis Adequacy 2006 Work Group (2006). Clinical practice guidelines for hemodialysis adequacy, update 2006. Am. J. Kidney Dis..

[10] Yevzlin A., Song G., Sanchez R., Becker Y. (2007). Fluoroscopically guided vs modified traditional placement of tunneled hemodialysis catheters: Clinical outcomes and cost analysis. J. Vasc. Access.

[11] Miller D.L., Balter S., Schueler B.A., Wagner L.K., Strauss K.J., Vano E. (2010). Clinical Radiation Management for Fluoroscopically Guided Interventional Procedures. Radiology.

[12] Romero J.R., Frey J.L., Schwamm L.H., Demaerschalk B.M., Chaliki H.P., Parikh G., Burke R.F., Babikian V.L. (2009). Cerebral ischemic events associated with ’bubble study’ for identification of right to left shunts. Stroke.

[13] Bassett G., Lin J., Tran M., Sistino J. (2014). Evaluating the potential risks of bubble studies during echocardiography. Perfusion.

